# Next‐generation bromodomain inhibitors of the SWI/SNF complex enhance DNA damage and cell death in glioblastoma

**DOI:** 10.1111/jcmm.17907

**Published:** 2023-08-18

**Authors:** Chuanhe Yang, Yali He, Yinan Wang, Peter J. McKinnon, Vijay Shahani, Duane D. Miller, Lawrence M. Pfeffer

**Affiliations:** ^1^ Department of Pathology and Laboratory Medicine College of Medicine, University of Tennessee Health Science Center Memphis Tennessee USA; ^2^ Department of Pharmaceutical Sciences College of Pharmacy, University of Tennessee Health Science Center Memphis Tennessee USA; ^3^ St. Jude Children's Research Hospital Memphis Tennessee USA; ^4^ Recursion Pharmaceuticals Inc Toronto Ontario M5V 2A2 Canada; ^5^ The Center for Cancer Research University of Tennessee Health Science Center Memphis Tennessee USA

**Keywords:** BRG‐1, BRM, bromodomain, cell death, DNA damage, glioblastoma, small molecule inhibitor, SWI/SNF

## Abstract

Glioblastoma (GBM) is an aggressive brain cancer with a poor prognosis. While surgical resection is the primary treatment, adjuvant temozolomide (TMZ) chemotherapy and radiotherapy only provide slight improvement in disease course and outcome. Unfortunately, most treated patients experience recurrence of highly aggressive, therapy‐resistant tumours and eventually succumb to the disease. To increase chemosensitivity and overcome therapy resistance, we have modified the chemical structure of the PFI‐3 bromodomain inhibitor of the BRG1 and BRM catalytic subunits of the SWI/SNF chromatin remodelling complex. Our modifications resulted in compounds that sensitized GBM to the DNA alkylating agent TMZ and the radiomimetic bleomycin. We screened these chemical analogues using a cell death ELISA with GBM cell lines and a cellular thermal shift assay using epitope tagged BRG1 or BRM bromodomains expressed in GBM cells. An active analogue, IV‐129, was then identified and further modified, resulting in new generation of bromodomain inhibitors with distinct properties. IV‐255 and IV‐275 had higher bioactivity than IV‐129, with IV‐255 selectively binding to the bromodomain of BRG1 and not BRM, while IV‐275 bound well to both BRG1 and BRM bromodomains. In contrast, IV‐191 did not bind to either bromodomain or alter GBM chemosensitivity. Importantly, both IV‐255 and IV‐275 markedly increased the extent of DNA damage induced by TMZ and bleomycin as determined by nuclear γH2AX staining. Our results demonstrate that these next‐generation inhibitors selectively bind to the bromodomains of catalytic subunits of the SWI/SNF complex and sensitize GBM to the anticancer effects of TMZ and bleomycin. This approach holds promise for improving the treatment of GBM.

## 1 INTRODUCTION

Gliomas are the most common primary brain cancers in adults. While Grade IV glioma (GBM, glioblastoma) is the most aggressive and deadliest brain tumour, Grade I glioma is the least malignant glioma. The primary treatment modality for GBM is surgical resection combined with adjuvant temozolomide (TMZ) chemotherapy and radiation therapy, which only provides slight improvement in disease course and outcome.^1^ The median time for GBM recurrence after surgery is 7 months and overall prognosis is dismal with a 5‐year survival of only 5%.^2^

The mammalian ATP‐dependent chromatin remodelling SWI/SNF complex, is an evolutionarily conserved multi‐subunit complex that regulates gene expression, differentiation, DNA repair and development.^3^ The two catalytic subunits, BRM (Brahma) and BRG1 (Brahma‐related gene 1) reposition and/or remodel nucleosomes, which opens or closes chromatin to regulate gene transcription.^4^ In adult glioma, BRG1 expression increases with histological tumour grade with the highest levels found in GBM patients. In contrast, BRM expression is inversely related to tumour grade with the lowest expression found in GBM patients. BRG1 functions as a tumour suppressor in cancers of the lung, ovaries, skin and blood with silencing or loss‐of‐function mutations enriched.^5^, ^6^, ^7^, ^8^, ^9^ In contrast, BRG1 has tumour promoting activity in several other cancers, including GBM.^7^, ^10^ Mutations of BRG1 are rarely found in multiple genomic databases of GBM patients.^11^ Moreover, we demonstrated that high BRG1 expression selectively localizes in GBM patient tumour tissue.^12^

BRG1 contains an evolutionarily conserved bromodomain protein–protein interaction module that binds acetyl‐lysine on protein and histone tails.^13^, ^14^ For example, BET bromodomain‐containing proteins regulate the expression of key oncogenes, and specific and potent BET inhibitors are now in cancer clinical trials.^15^, ^16^, ^17^ Thus, bromodomains are attractive targets in cancer, yet no inhibitors selective for BRG1 have been identified. PFI‐3 was developed as a highly selective small molecule bromodomain inhibitor of the BRG1 and BRM subunits of the SWI/SNF complex, which has minimal ‘off‐target’ effects in primary human cells and no evidence of toxicity on the NCI‐60 panel of tumour cell lines.^18^, ^19^ We found that, although PFI‐3 does not affect GBM cell proliferation, PFI‐3 increased the sensitivity of various established GBM cell lines to TMZ as well as overcame TMZ‐resistance.^12^, ^20^

In the present study, we have developed active second‐generation bromodomain inhibitors, denoted therapy enhancing drugs (TEDs), by making modifications to the chemical structure of PFI‐3. As described herein, we identified TEDs that markedly enhanced the sensitivity of GBM cells to DNA damaging agents. In addition, we identified one TED that was highly selective for binding to the BRG1 subunit of the SWI/SNF complex. Importantly, it significantly enhances sensitivity of GBM to DNA damaging agents in BRG1 expressing GBM cells but not in BRG1 knockout GBM cells.

## 2 MATERIALS AND METHODS

### 2.1 Synthesis of TEDs

**FIGURE 1:**
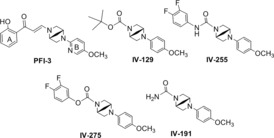
Structure of PFI‐3 and new analogues IV‐129, IV‐191, IV‐255 and IV‐275.

Utilising PFI‐3 as a lead compound, novel TEDs were designed and synthesized, which were expected to enhance the sensitivity of GBM cells to DNA alkylating agents. As illustrated in Figure 1, we aimed to optimize the bromodomain inhibitory activity by eliminating the double‐bond and modifying the aromatic A‐ring and B‐ring of the structure. This approach enabled us to gain a better understanding of the structure activity relationship of these bromodomain inhibitors and helped us to design analogues with improved biological activity.

### 2.2 Biological reagents and cell cultures

MT330 (Department of Neurosurgery, UTHSC) and T98G, U87 and LN229 (ATCC) GBM cell lines were grown in DMEM containing 10% foetal bovine serum (Hyclone) supplemented with penicillin (100 IU/mL) and streptomycin (100 μg/mL) at 37°C with 5% CO_2_. The cells were authenticated by short tandem repeat analysis.

### 2.3 Cell death, viability and invasion assays

For apoptosis assays, cells were plated into 48‐well plates (1 × 10^4^ cells/well), and after 3 days of drug treatment the levels of apoptosis in the attached cells were determined according to the instructions using the cell death ELISA^PLUS^ assay (Roche), which measures cytoplasmic histone‐associated DNA fragments.^21^ In addition, to determine the effect of various drugs on cell viability and death, the Live/Dead cell viability/cytotoxicity assay (Molecular Probes) as previously described.^22^ Images were captured on a Zeiss LSM700 laser scanning confocal microscope. Invasion assays using transwell inserts (BD Biosciences) were performed as previously described.^23^

### 2.4 Cellular thermal shift assay (CETSA)

The binding activity of PFI‐3 and TEDs to the bromodomain of the BRG1 and BRM subunits of SWI/SNF was assessed by previously described CETSA.^24^ In brief, MT330 GBM cells transduced with an epitope tagged BRG1 or BRM bromodomain were treated with PFI‐3 (30 μM), the indicated TEDs (30 μM) or DMSO as a vehicle control for 3 h. After heating over a temperature range from 44.5 to 55.6°C for 5 min, the cells were lysed, placed on ice at 4°C and then immunoblotted for BRG1, BRM or actin.

### 2.5 Gene expression analysis

Total RNA was extracted using RNeasy mini kits (Qiagen Inc.). Quantitative real time PCR (qPCR) was performed using gene‐specific primers for CXCL11 (forward 5′ GACGCTGTCTTTGCATAGGC and reverse 3′ GGATTTAGGCATCGTTGTCCTTT), IRF7 (forward 5′ GCTGGACGTGACCATGTA and reverse 3′ GGGCCGTATAGGAACGTGC), STAT1 (forward 5′ CAGCTTGACTCAAAATTCCTGGA and reverse 3′ TGAAGATTACGCTTGCTTTTCCT), IRF1 (forward 5′ CTGTGCGAGTGTACCGGATG and reverse 3′ ATCCCCACATGACTTCCTCTT), MX1 (forward 5′ TCCCACCCTCTATTACTGAATGG and reverse 3′ GGGAAGGGCAACTCCTGAC) and BETA‐ACTIN (forward 5′‐GGACTTCGAGCAAGAGATGG‐3′ and reverse 3′AGCACTGTGTTGGCGTACAG‐3′) with an iScript one‐step RT‐PCR kit containing SYBR Green (Bio‐Rad). The reaction parameters were as follow: cDNA synthesis at 50°C for 10 min, transcriptase inactivation at 95°C for 5 min, and PCR cycling at 95°C for 10 s and 60°C for 30 s for 40 cycles. Gene expression was normalized relative to actin expression.

### 2.6 Generation of BRG1 and BRM knockout cells

Lentiviral CRISPR/Cas9‐mediated BRG1 and BRM knockout vectors were constructed by cloning three different guide RNAs into the lentiviral vector pLenti CRISPR V2 as previously described.^25^ Control vector was constructed by inserting the EGFP gRNA sequence into the lentiviral vector. Lentivirus were packaged in 293FT cells. Stable pools of BRG1^KO^ and BRM^KO^ cells were generated by transducing GBM cells with the lentiviral CRIPSR/Cas9 vectors and selected with 3 μg/mL puromycin. After selection, BRG1^KO^ and BRM^KO^ cells were expanded in the absence of puromycin and subsequently used in experiments.

### 2.7 Immunostaining for γH2AX

GBM cells grown on 8‐well glass chamber slides (Millipore) were treated with TEDs (20 μM) with or without TMZ (200 μM) for 48 h. Slides were washed with PBS and then fixed with 4% paraformaldehyde/PBS containing 0.3% Triton‐X100 for 30 min at 25°C. After blocking with 10% goat serum and 1% BSA/PBS for 1 h, the slides were incubated with the indicated primary γH2AX antibody overnight at 4°C. After incubating with goat anti‐rabbit (or rabbit anti‐mouse) Alexa Fluor 488 secondary antibody at 25°C for 90 min, DNA was counterstained with DAPI (Vectra Laboratories). Images were captured on a Zeiss LSM700 laser scanning confocal microscope.

### 2.8 Statistical analyses

At least two independent experiments were performed in duplicate, and data are presented as means ± standard deviation (S.D.). Data were analysed by anova (Analysis of Variance) and post‐hoc least significant difference analysis or Student's *t*‐tests.

### 2.9 Ethics Statement

Not applicable.

## 3 RESULTS

### 3.1 The design and synthesis of PFI‐3 analogues

As illustrated in Figure 1, we aimed to improve the activity of PFI‐3 through major structural changes to optimize the bromodomain inhibitory activity by replacing acryloyl (3‐oxo‐prop‐1‐enyl) moiety with an ester or amide moiety between the A ring and the N atom of bicyclic ring system. We have changed the substituents on both the aromatic A and B‐ring of PFI‐3 to give the resulting analogues illustrated in Figure 1. Our approach enabled us to gain a better understanding of the structure activity relationship of these bromodomain inhibitors and help us to design better analogues for enhancing the action of TMZ in treating GBM. We confirmed the structure of the new analogues using nuclear magnetic resonance and mass spectrometry, and their purity was confirmed by high performance liquid chromatography.

### 3.2 TEDs enhance the apoptosis‐inducing activity of TMZ in GBM cells

**FIGURE 2:**
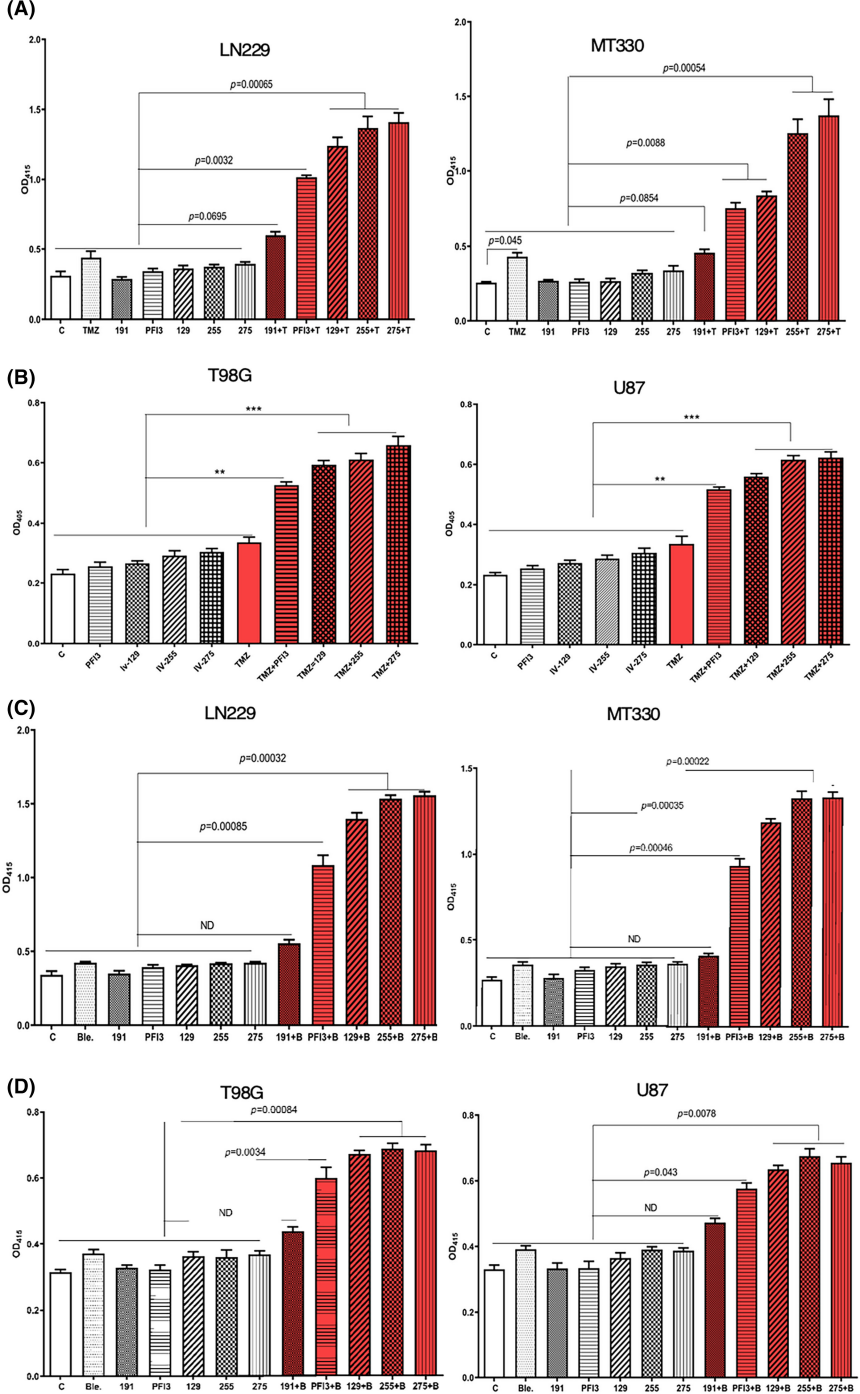
PFI‐3 and TEDs enhance apoptosis induced by TMZ and bleomycin. (A, B) MT330 and LN229 (A) or U87 and T98G (B) GBM cells were treated with PFI‐3 or the indicated TEDs alone (10 μM) and with TMZ (200 μM). (C,D) MT330 and LN229 (C) or T98G and U87 (D) cells were treated with PFI‐3 or the indicated TEDs alone (10 μM), and bleomycin (5 μM). Control cells were treated with vehicle (DMSO). Apoptosis was determined with a cell death ELISA on cells attached to the plates at 72 h, which quantifies histone‐complexed DNA fragments. Calculated *p*‐values are shown.

The DNA damaging agent TMZ is frontline therapy for patients with GBM, and we previously found that the BRG1/BRM bromodomain inhibitor PFI‐3 can enhance the sensitivity of GBM cells to the anticancer activity of TMZ in vitro.^25^ Additional chemical modifications of the PFI‐3 structure resulted in our lead molecule IV‐129 (Figure 1) that had greater activity than PFI‐3 to enhance the sensitivity of MT330 and LN229 GBM cells to the apoptosis‐inducing activity of 200 μM TMZ (Figure 2A), which was denoted as a TED. Further refinements of the IV‐129 structure resulted in the generation of TEDs IV‐255 and IV‐275 that had even greater bioactivity in cell death assays in both GBM cell lines with TMZ (Figure 2A). It was of particular interest that TED IV‐191 had no effect on the induction of apoptosis by TMZ in both GBM cell lines.

### 3.3 TEDs enhance the sensitivity of TMZ‐resistant GBM cells to the apoptosis‐inducing activity of TMZ

To further evaluate the ability of TEDs to enhance TMZ activity, we used two GBM cell lines (T98G and U87) that are highly resistant to the cell death‐inducing activity of TMZ. While IV‐129 was found to be much more potent than PFI‐3 in sensitising U87 and T98G GBM cells in vitro to the cell‐death inducing effects of TMZ (Figure 2B), both IV‐255 and IV‐275 were more potent than IV‐129 in enhancing TMZ‐induced cell death.

### 3.4 TEDs also sensitize GBM cells to bleomycin‐induced cell death

Bleomycin is a chemotherapeutic drug that is used to treat various cancers. In addition, bleomycin induces DNA damage in a manner similar to that of ionising radiation^26^ and it has been used in previous studies as an in vitro radiomimetic DNA‐cleaving agent. We then examined the ability of TEDs to enhance the cell death inducing activity of bleomycin. Consistent with our other studies, none of the TEDs (IV‐129, IV‐255 and IV‐275) alone had any death inducing activity in GBM cells. While a low dose of bleomycin (5 μM) induced very low levels of cell death in either TMZ‐sensitive (Figure 2C) or TMZ‐resistant (Figure 2D) GBM cells, bleomycin in combination with TEDs (IV‐255 and IV‐275) had a marked effect on GBM cell death. Furthermore, as was the case for TMZ treatment, IV‐191 did not sensitive GBM cells to bleomycin‐induced cell death. Taken together these results indicate that these two TEDs, IV‐255 and IV‐275, can potently enhance TMZ‐induced cell death in TMZ‐sensitive GBM cells, and partially overcome TMZ‐resistance in GBM cells.

### 3.5 The binding of TEDs to the bromodomain of BRG1 and BRM

**FIGURE 3:**
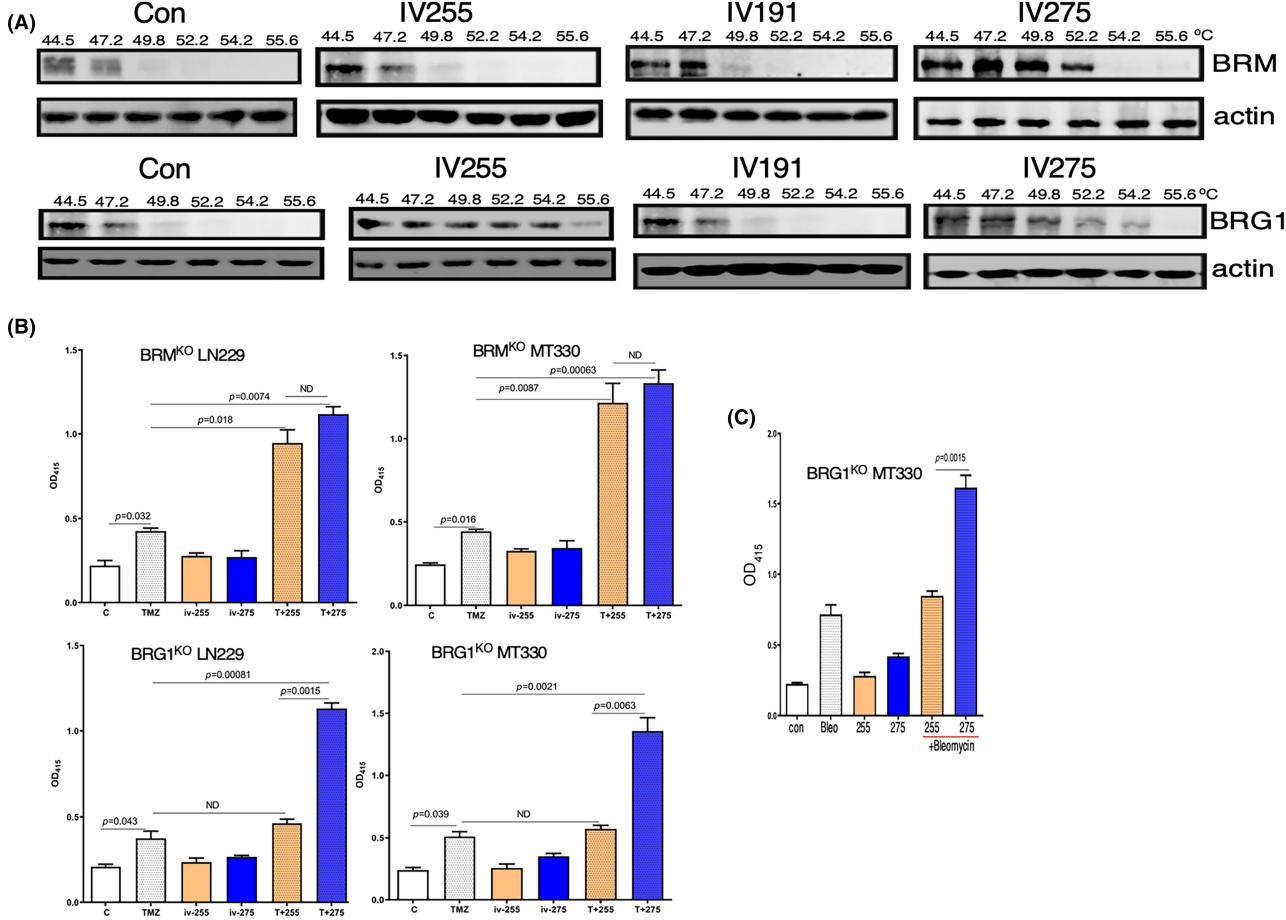
TED IV‐255 selectively binds to the BRG1 bromodomain and is required for BRG1 to enhance TMZ‐ or bleomycin‐induced apoptosis. (A) For the CETSAs, MT330 cells expressing the BRG1 epitope tagged bromodomain construct were treated with either PFI‐3 or TEDs (30 μM) for 3 h. Cells were treated with vehicle (DMSO) as a control. After heating over a temperature range from 44.5 to 55.6°C for 5 min, the cells were lysed, placed on ice at 4°C and then immunoblotted for BRG1 or Actin. (B, C) BRG1^KO^ or BRM^KO^ LN229 and MT330 GBM cells were treated with (B) TMZ (100 μM) or (C) bleomycin (5 μM) for 72 h and apoptosis was determined by a cell death ELISA as described in Figure 2. Calculated *p*‐values are shown.

The binding of the TEDs to the BRG1 and BRM bromodomains was assessed by the cellular thermal shift assay (CETSA), which measures thermostability of these bromodomains when complexed to the TEDs.^24^, ^25^ In brief, MT330 cells that express a BRG1 or BRM bromodomain construct were treated with TEDs for 2 h. After heating for 5 min over a temperature range from 44.5 to 55.6°C, cells were lysed and immunoblotted for BRG1 or BRM. As shown in Figure 3A, YH‐IV‐255 and YH‐IV‐275 significantly increased the thermostability of the BRM bromodomain with the band becoming nearly undetectable at 55.6°C in both IV‐255 and IV‐275 treated cells, while the band disappeared at 49.8°C in the vehicle control. However, IV‐275 significantly increased the thermostability of the BRM bromodomain, while IV‐255 had no effect since the thermal shift of the IV‐255/BRM complex was similar to the vehicle control. These results indicate that IV‐255 is a selective binder to the BRG1 bromodomain. In contrast to the results with IV‐255 and IV‐275, YH‐IV‐191 did not stabilize complex formation with either the BRG1 or BRM bromodomain. Therefore, although IV‐191 is chemically similar to the other TEDs, it does not bind to either bromodomain, which is also consistent with IV‐191 not enhancing the cell death‐inducing activity of TMZ or bleomycin (Figure 2).

### 3.6 The identification of IV‐255 as a BRG1‐selective TED

Thermal shift assays show that IV‐255 binds to the BRG1 bromodomain but does not bind the BRM bromodomain (Figure 3A), suggesting that IV‐255 has specificity for the BRG1 bromodomain. GBM cell lines with either BRG1 or BRM knocked out were generated by CRISPR/Cas9 gene editing, and the knockout was validated by immunoblotting.^25^ We then determined whether ablating expression of these subunits altered the enhancing activity of TEDs in GBM cells to TMZ‐induced cell death. While IV‐255 and IV‐275 have very similar activity in enhancing TMZ‐induced cell death in BRM^KO^ MT330 and LN229 GBM cells, IV‐255 has no such effect in BRG1^KO^ GBM cells (Figure 3B). These results are consistent with our finding that IV‐255 selectively binds the BRG1 bromodomain and can only enhance TMZ‐induced death in GBM cells that express the BRG1 subunit. Furthermore, similar to the results with TMZ, while parental LN229 cells are sensitive to the death‐inducing activity of bleomycin, treatment of BRG1^KO^ MT330 cells with IV‐255 does not result in bleomycin‐induced cell death, while treatment with IV‐275 does enhance bleomycin‐induced MT330 cell death. Taken together we have identified IV‐255 as a selective small molecule inhibitor of the BRG1 bromodomain. In contrast, IV‐275, which only differs by a single chemical substitution from IV‐255, is an inhibitor of both bromodomains.

### 3.7 TEDs increase the amount of DNA‐damage induced by TMZ and bleomycin

**FIGURE 4:**
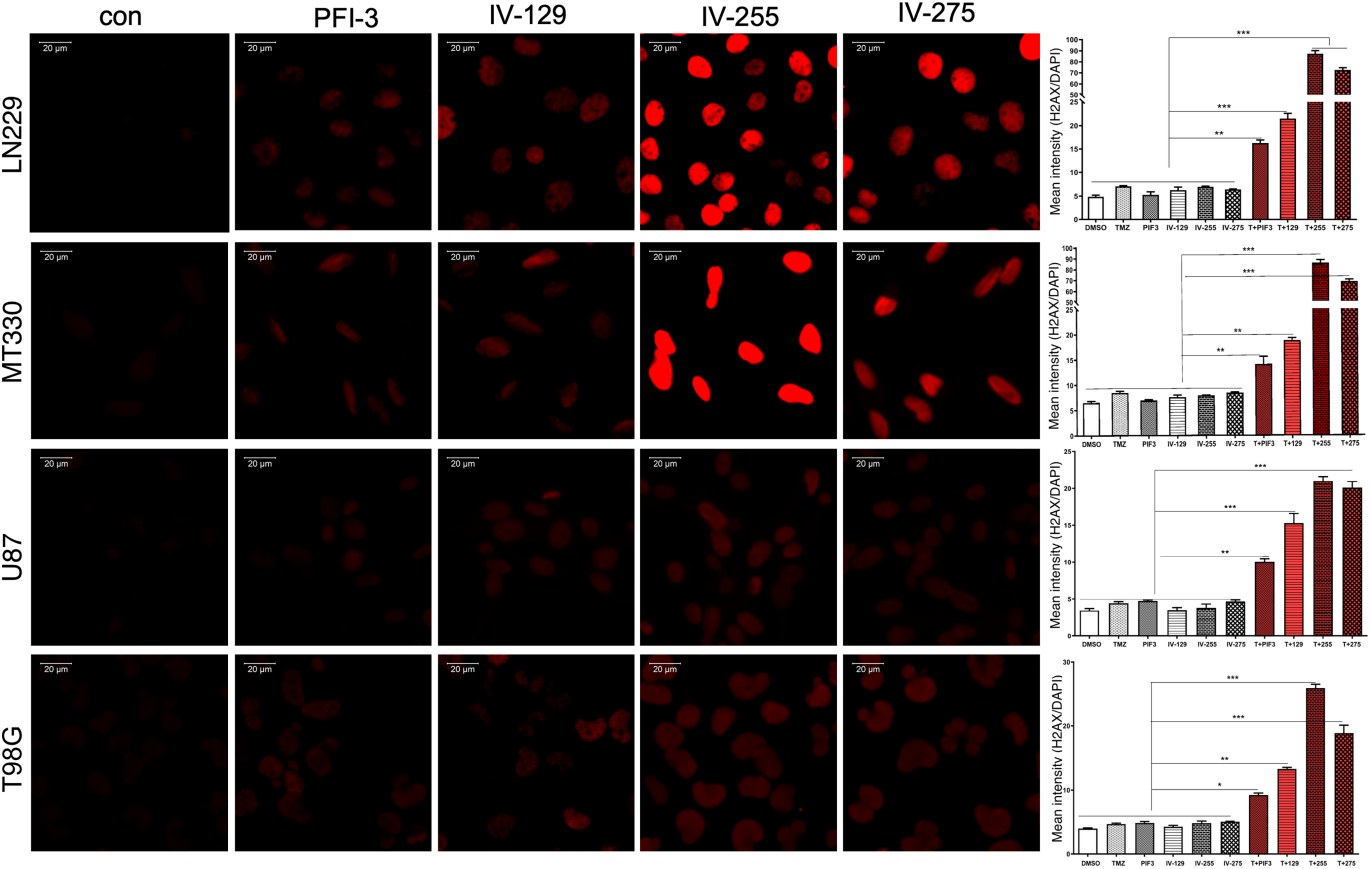
PFI‐3 and TEDs sensitized GBM cells to TMZ‐induced DNA damage. MT330 cells were treated with TMZ (200 μM) and PFI‐3 or TEDs (20 μM). After 48 h of treatment, cells were fixed and immunostained for γH2AX. Immunostaining analysis was performed on a Zeiss LSM 700 confocal microscope and data analysed with ZEN software. The graphs represent the ratio of γH2AX versus DAPI staining. (*p < 0.05, **p < 0.01 and ***p < 0.005).

Both TMZ and bleomycin are chemotherapeutic agents that induce DNA damage and promote subsequent tumour cell death. TMZ alkylates DNA at the N‐7 or O‐6 positions of guanine residues, which damages the DNA and causes DNA strand breaks. Bleomycin oxidatively damages DNA by binding to metal ions and forming complexes, which causes DNA strand breaks.^26^ To investigate the mechanism that underlies the ability of TEDs to enhance GBM cell death induced by TMZ and bleomycin, we examined the effects of TEDs to induce DNA damage as evidenced by immunostaining of GBM cells for γH2AX, which is the phosphorylated form of histone H2AX and functions as a sensitive marker for DNA double‐strand breaks.^27^ While TMZ alone induces slight increases in γH2AX immunostaining in LN229 and MT330 TMZ‐sensitive cells, treatment with PFI‐3 or the TEDs in combination with TMZ caused a marked increase in the induction of DNA breaks as evidenced by γH2AX staining (Figure 4). Most importantly, both IV‐255 and IV‐275 caused a more marked effect on γH2AX staining in combination with TMZ. As expected TMZ alone had no effect on γH2AX staining in U87 and T98G TMZ resistant cells (Figure 4). In contrast, in the presence of PFI‐3 and TEDs γH2AX staining was increased in TMZ‐treated cells. To quantify the effect on γH2AX staining, we then determined the ratio of γH2AX staining relative to the DAPI nuclear counterstaining. As shown in the graphs in Figure 4, both IV‐255 and IV‐275 caused a marked increase in the extent of DNA‐damage upon TMZ treatment in both TMZ‐sensitive (LN229 and MT330) and TMZ‐resistant (U87 and T98G) GBM cell lines.

### 3.8 TEDs enhance the effect of TMZ on reducing GBM cell viability

**FIGURE 5:**
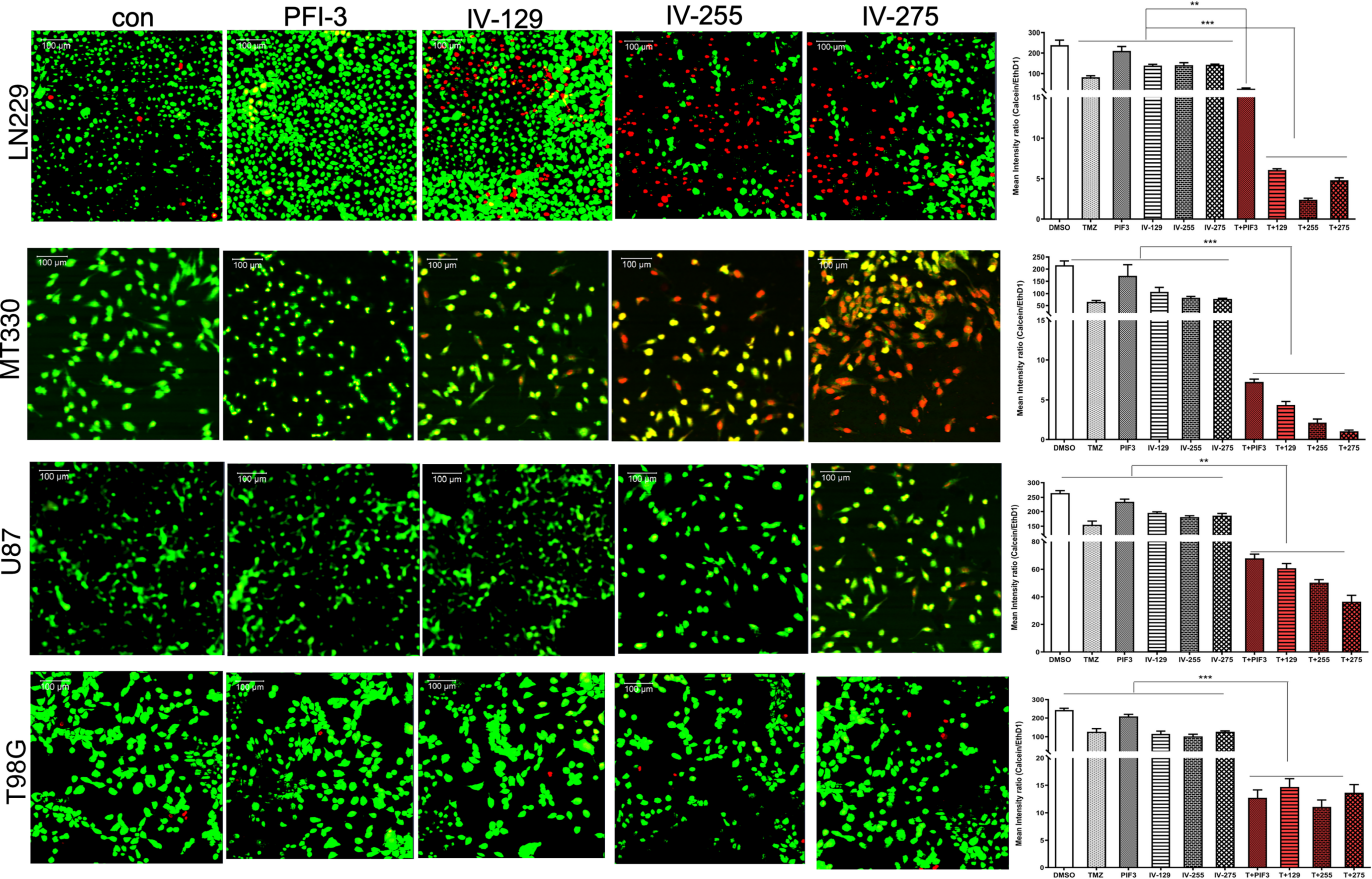
PFI‐3 and TEDs promote TMZ‐induced reduction of GBM cell viability. MT330 cells were treated with TMZ (200 μM) and PFI‐3 or TEDs (20 μM). After 48 h of treatment, cells were analysed by LIVE/DEAD Viability assay and imaged on Zeiss LSM 700 confocal microscope. While live cells fluoresce bright green, dead cells fluoresce red‐orange. The graphs represent the ratio of live (calcein AM) versus dead (ethidium homodimer) staining. (*p < 0.05, **p < 0.01 and ***p < 0.005).

Since we found that TEDs increase DNA damage induced by TMZ, which should lead to increase in cell death, we next simultaneously determined live and dead cells with the live/dead cell fluorescent assay. Exposure of TMZ‐sensitive (LN229 and MT330) and TMZ‐resistant (U87 and T98G) GBM cell lines to TEDs or sublethal doses of TMZ did not induce significant cell death in any of the GBM cells examined. In contrast, combined treatment of TMZ with TEDs IV‐255 and IV‐275 markedly increased the number of dead (red) cells and reduced the number of live (green) cells in cultures of TMZ‐sensitive and TMZ‐resistant GBM cell lines (Figure 5). Only a few live cells are observed after TMZ treatment of IV‐255 and IV‐275 TMZ‐sensitive (LN229 and MT330) cells. It is important to note that the reduction in cell viability is more marked upon TMZ treatment of the more TMZ‐sensitive LN229 and MT330 relative to that of the TMZ‐resistant U87 and T98G cell lines (Figure 5).

### 3.9 TEDS inhibit the invasiveness of GBM cells

**FIGURE 6:**
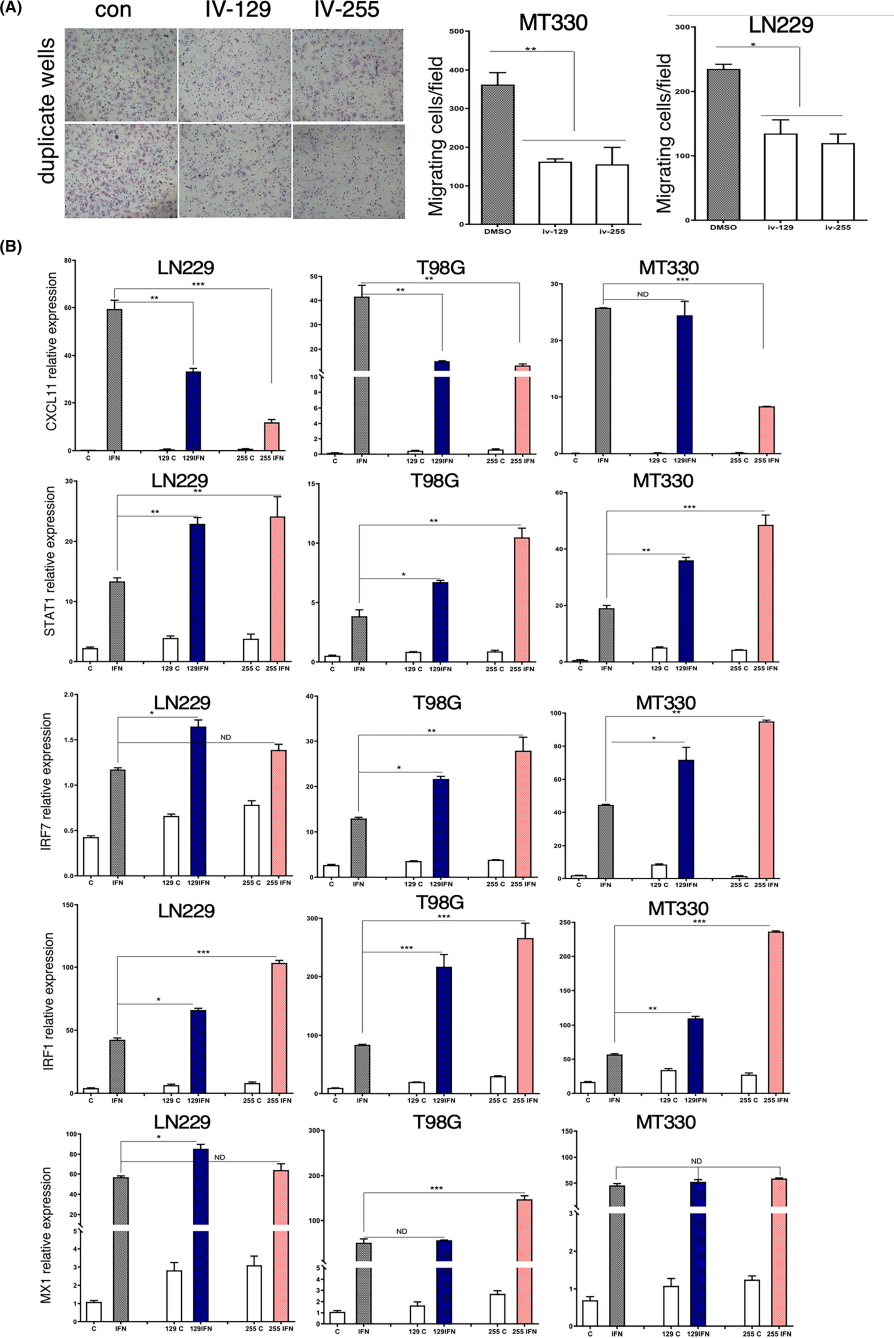
The effects of TEDs on cell invasion and IFN‐induced gene expression in GBM cells. (A) MT330 and LN229 cells were treated with IV‐129 or IV255 (20 μM) for 24 h, and invasion was determined by transwell assays. (B) RNA was prepared from MT330, LN229 and T98G GBM cells which were treated for 24 h with TEDs in the presence or absence of IFNαCon1 (1000 U/mL). The expression of the indicated genes was determined by qPCR and normalized to β‐actin expression. (*p < 0.05, **p < 0.01 and ***p < 0.005).

The GBM cells are intrinsically high invasive, which plays an important role in GBM pathogenicity. Thus, based on our previous studies that the BRG‐1 subunit plays a pro‐oncogenic role in GBM,^25^ we investigated the effect of TEDs on the invasiveness of GBM cells. Treatment of both MT330 and LN229 GBM cells with the TEDs, IV‐129 and IV‐255, resulted in a significant inhibition of cell invasion (Figure 6A).

### 3.10 The effect of TEDs on IFN‐regulated gene expression

In previous studies, we found that PFI‐3 had a significant inhibitory effect on IFN‐stimulated gene (ISG) expression.^28^ Based on our finding that these newly developed TEDs were more potent inhibitors of the BRG1/BRM bromodomain than PFI‐3, we next examined the effects of IV‐129 and IV‐255 on basal and IFN‐induced ISG expression. As expected, IFN robustly induced the expression of the ISGs, *CXCL11, STAT1, IRF7, IRF1 and MX1*, in all the GBM cell lines we examined. While treatment with TEDs reduced the IFN‐induced expression of *CXCl11*, TEDs increased IFN‐induced expression of *STAT1, IRF1* and *IRF7*. In contrast, although IFN induced a significant increase in MX1, TEDs did not alter IFN‐induced MX1 expression. It is important to note that in gene expression assays IV‐255 tended to have greater effects on IFN‐induced ISG expression as compared to IV‐129, consistent with our findings of enhanced activity in other bioassays performed herein. Furthermore, although T98G and U87 are TMZ‐resistant GBM cell lines (Figure 6B and data not shown), the effects of the TEDs on ISG expression were similar to that of the TMZ‐sensitive LN229 and MT330 GBM cells.

### 3.11 The drug‐like properties of TEDs

Using Cyclica's Ligand Express™ platform, several key chemical/biological properties of TEDs (PFI‐3, 129, 191, IV‐255 and IV‐275) were predicted. MatchMaker™, a deep‐convolutional neural network trained on protein structure and drug‐target interaction data, was used to predict the polypharmacology profile of TED molecules.^29^ Specifically, the critical off‐target interaction with BRG1 and BRM was assessed against the background of the structurally characterized human proteome (8504 proteins). PFI‐3 and TEDs possessed good rank percentiles for these two targets with PFI‐3 and IV‐129 having a slightly increased likelihood of binding. Consistently across all TEDs, BRG1 ranked higher than BRM, which suggests compounds have an increased likelihood of interaction for BRG1. Furthermore, these results were further validated by a virtual molecular docking program using this platform.

Within the Ligand Express platform, the Pareto‐Optimal Embedded Modelling (POEM) algorithm was then used to predict the activity of TEDs in key physicochemical models.^30^ A POEM classifier model for blood brain barrier penetration predicted that all TEDs would pass the barrier (0.87–0.99), they were likely not to be a substrate of P‐glycoprotein (0.13–0.40), and that all were likely to be absorbed within the intestine (0.92–0.99). A POEM model assessing likelihood of Ames mutagenicity (0.44–0.60) did not classify compounds as mutagenic, with predictions near a random value of 0.5. TEDs were assessed using POEM toxicity‐related proteins classification models, including the cannabinoid receptor 2 and androgen receptor. TEDs IV‐129, IV‐255 and IV‐275 were classified as non‐binders of cannabinoid receptor 2 (0.11–0.33), with PFI‐3 and IV‐191 having predictions close to random. TEDs IV‐255, IV‐275 and PFI‐3 were weakly predicted to be non‐binders of androgen receptor (0.28–0.32), with IV‐129 and IV‐191 with POEM values close to random. A regression‐based POEM model for aqueous solubility, predicted all compounds to be within an order of magnitude to PFI‐3, suggesting that all would have comparable solubility. Thus, these physicochemical properties bode well for the potential development of these compounds as candidate therapeutic agents going forward.

## 4 DISCUSSION

The SWI/SNF chromatin remodelling complex binds to the promoters and enhancers of specific genes to regulate cell proliferation, differentiation, metabolism and DNA repair.^31^, ^32^, ^33^, ^34^, ^35^, ^36^ Genomic alterations of the BRG1 and BRM subunits of SWI/SNF in several human malignancies is consistent with their function as tumour suppressors in these cancers.^3^, ^11^ However, in many cancers neither BRG1 nor BRM are mutated but instead they are overexpressed, thus suggesting that they may also play a pro‐tumorigenic role.^12^, ^20^, ^37^ We recently found that found that BRG1 expression was higher in GBM tumour tissue compared to non‐tumour tissue, while BRM was expressed at lower levels.^25^ Gene deletion studies and knockdown studies on BRG1 in GBM cells suggest that BRG1 a pro‐tumorigenic role in GBM, and that BRG1 may be an attractive therapeutic target in GBM.

PFI‐3 was developed as a small‐molecule inhibitor that binds and inhibits the function of the bromodomains of BRG1 and BRM.^19^, ^38^ We found that, although PFI‐3 alone does not alter cell proliferation, PFI‐3 enhanced GBM cells sensitivity to the DNA alkylating TMZ.^12^, ^20^, ^24^ Herein, we described the development of a new generation of structural analogues that further enhance GBM cell sensitivity to TMZ, and they are thus denoted TEDs. While the two most potent TEDs IV‐255 and IV‐275 bound to the bromodomain of BRG1, IV‐255 did not bind to the BRM bromodomain, while IV‐275 did bind as determined by CETSA. Moreover, we show that, although IV‐255 and IV‐275 enhanced TMZ‐induced death in BRM^KO^ GBM cells, IV‐255 did not have activity in BRG1^KO^ cells. Taken together these results demonstrate that IV‐255 is a small molecule inhibitor of the BRG1 bromodomain. Interestingly, we also found that another structural analogue of PFI‐3, did not bind to either bromodomain or enhance the sensitivity of GBM cells to TMZ. These data indicate that, although this compound is structurally similar to other potent TEDs, IV‐191 is an inactive analogue. Moreover, we found that TEDs enhance the sensitivity of GBM cells to bleomycin that is often used as a radiomimetic, suggesting that TEDs may also be radiosensitizers as well as chemosensitizers.

We also examined the mechanism whereby TEDs enhance TMZ‐induced cell death in GBM cells. TMZ, like other alkylating agents, induce DNA damage in cells which subsequently results in cancer cell death. We found that TEDs markedly increased the number of cells stained by γH2AX as well as the extent of staining in GBM cells induced by TMZ, thus indicating that TEDs enhanced DNA damage induced by TMZ. BRG1 promotes DNA strand breaks through nucleosome repositioning and altered recruitment of repair factors.^39^, ^40^ The BRG‐1/BRM inhibitor PFI‐3 exerts its DNA‐sensitising effects of doxorubicin in cancer cells by inhibiting DNA repair.^41^ In contrast, the results described herein suggest that TEDs increase the amount of DNA breaks induced by TMZ and bleomycin. Moreover, consistent with our cell death ELISA studies, we show that TEDs markedly decrease the number of viable GBM cells and increase the number of dead GBM cells using live/dead cell assays.

Furthermore, consistent with BRG1 playing a pro‐tumorigenic role in GBM, we found that TEDs decrease GBM cancer cell invasion. This effect has therapeutic importance since high invasiveness is a hallmark of GBM. We previously found that BRG1 selectively regulated the expression of several well‐known ISGs in GBM cells,^12^ which is not surprising as BRG1 is a catalytic subunit of the SWI/SNF complex remodelling complex. SWI/SNF inhibition would be expected to inhibit the nucleosome remodelling and downstream chromatin changes required to activate many genes. The activation of a subset of ISGs, while less straightforward to understand mechanistically, may be due to the loss of expression of an ISG transcriptional repressor.^42^ The role of the SWI/SNF complex in promoting ISG transcription has been the focus of several earlier studies.^43^, ^44^ Moreover, PFI‐3 at higher concentrations than we used was found to decrease the expression of several genes.^28^ In contrast, we show herein that TEDs increased the expression of ISGs such as STAT1, IRF1 and IRF7. Nonetheless consistent with these earlier studies TEDs decreased the expression of CXCL11. Previously we found that PFI‐3 inhibited ISG activation,^28^ which is consistent with our findings of the inhibitory effects of TEDs on STAT1, IRF1 and IRF7 expression. The selective effect of TEDs on gene expression was also borne out by the finding that the ISG MX1 was unaffected by TEDs. The effect of TEDs on ISG expression was observed in both TMZ‐resistant and TMZ‐sensitive GBM cells, indicating that the gene expression effects of TEDs are not related to the pathway of TMZ‐induced DNA damage. It remains presently unknown how TEDs selectively regulate ISG expression.

Taken together, we found that TEDs selectively bind to the bromodomains of catalytic subunits of the SWI/SNF complex and sensitize GBM to the anticancer effects of TMZ in vitro. A limitation of the present study is that the anticancer effects of TEDs have not been tested in animal GBM models in vivo, which will be an important focus of future studies.

## AUTHOR CONTRIBUTIONS

**Chuan He Yang:** Conceptualization (supporting); data curation (lead); formal analysis (lead); investigation (lead); methodology (lead); validation (equal); visualization (lead); writing – original draft (supporting); writing – review and editing (supporting). **Yali He:** Conceptualization (supporting); data curation (lead); investigation (lead); methodology (lead); resources (lead); writing – original draft (supporting); writing – review and editing (supporting). **Yinan Wang:** Conceptualization (supporting); data curation (lead); formal analysis (supporting); investigation (lead); writing – original draft (supporting); writing – review and editing (supporting). **Peter McKinnon:** Methodology (lead); resources (lead); writing – original draft (supporting); writing – review and editing (supporting). **Vijay Shahani:** Data curation (lead); formal analysis (lead); investigation (lead); methodology (lead); resources (lead); software (lead); writing – original draft (supporting); writing – review and editing (supporting). **Duane Miller:** Conceptualization (lead); formal analysis (lead); funding acquisition (lead); methodology (lead); project administration (lead); resources (lead); supervision (lead); validation (lead); writing – original draft (lead); writing – review and editing (lead). **Lawrence Pfeffer:** Conceptualization (lead); data curation (supporting); formal analysis (lead); funding acquisition (lead); investigation (supporting); methodology (supporting); resources (lead); supervision (lead); validation (supporting); visualization (lead); writing – original draft (lead); writing – review and editing (lead).

## CONFLICT OF INTEREST STATEMENT

C.Y. Y.H., Y.W. D.D.M., and L.M.P. are inventors on the US Provisional Application 63/491,474 entitled Novel Bromodomain Inhibitors for Cancer Therapy, which covers the TEDs described herein. V.S. is an employee of Recursion Pharmaceuticals Inc., and may own stock in Recursion Pharmaceutical Inc. Recursion Pharmaceuticals owns and maintains MatchMaker. All authors declare no other conflicts of interest.

## Data Availability

The data that support the findings of this study and the BRG1^KO^ and BRM^KO^ GBM cells generated during and/or analysed during the current study are available from the corresponding author on reasonable request.
